# Recent advances in the microbial synthesis of lactate-based copolymer

**DOI:** 10.1186/s40643-021-00458-3

**Published:** 2021-10-22

**Authors:** Pengye Guo, Yuanchan Luo, Ju Wu, Hui Wu

**Affiliations:** 1 State Key Laboratory of Bioreactor Engineering, School of Biotechnology, East China University of Science and Technology, 130 Meilong Road, Shanghai, 200237 China; 2 Shanghai Collaborative Innovation Center for Biomanufacturing Technology, 130 Meilong Road, Shanghai, 200237 China; 3 Key Laboratory of Bio-Based Material Engineering of China National Light Industry Council, 130 Meilong Road, Shanghai, 200237 China

**Keywords:** Lactate-based copolymer, P(LA-*co*-3HB), Microbial synthesis, Production strategy

## Abstract

Due to the increasing environmental pollution of un-degradable plastics and the consumption of non-renewable resources, more attention has been attracted by new bio-degradable/based polymers produced from renewable resources. Polylactic acid (PLA) is one of the most representative bio-based materials, with obvious advantages and disadvantages, and has a wide range of applications in industry, medicine, and research. By copolymerizing to make up for its deficiencies, the obtained copolymers have more excellent properties. The development of a one-step microbial metabolism production process of the lactate (LA)-based copolymers overcomes the inherent shortcomings in the traditional chemical synthesis process. The most common lactate-based copolymer is poly(lactate-*co*-3-hydroxybutyrate) [P(LA-*co*-3HB)], within which the difference of LA monomer fraction will cause the change in the material properties. It is necessary to regulate LA monomer fraction by appropriate methods. Based on synthetic biology and systems metabolic engineering, this review mainly focus on how did the different production strategies (such as enzyme engineering, fermentation engineering, etc.) of P(LA-*co*-3HB) optimize the chassis cells to efficiently produce it. In addition, the metabolic engineering strategies of some other lactate-based copolymers are also introduced in this article. These studies would facilitate to expand the application fields of the corresponding materials.

## Introduction

Almost all traditional organic copolymers come from petrochemical industry, which will cause ‘greenhouse effect’, ‘white pollution’, and other environmental problems in the process of production, application, and management. With the increasing tension of resource development and people’s continued attention to the environmental issues, most of the research concepts in recent years are in line with the characteristics of recycling and environmental protection. Bio-based polymers, which can be degraded by microorganisms and are made from natural materials, have attracted much attention in recent years (Taguchi et al. 2008; Matsumoto and Taguchi 2010; Park et al. 2012a; Yang et al. 2013; Choi et al. 2020b).

Nowadays, PLA is one of the most representative bio-based polymers, its most notable features are biocompatibility and biodegradability (Pang et al. 2010; Shah et al. 2014). In addition to the above mentioned, PLA also has pros and cons, such as the advantages of high strength, high modulus, biocompostability, low toxicity, etc., as well as the disadvantages of hydrophobicity, low impact toughness, etc. Due to its complex biological and chemical production processes, PLA is relatively expensive compared with other commercial plastics (Lee et al. 2019; Singhvi et al. 2019). In order to make up for its shortcomings, copolymerization with other compositions is the most effective method. After copolymerization, the properties of PLA will be adjusted, such as degradation cycle, mechanical properties, hydrophilic properties, lipophilic properties, etc. At the same time, with the change of the composition and the proportion of the copolymers, PLA and its copolymers [belonging to polyhydroxyalkanoate (PHA), which is the general term for a class of bio-based polymers] will have more extensive applications, such as medical field (efficient nanocarriers for drug delivery applications, etc.) and other fields (Södergård and Stolt 2002; Makadia and Siegel 2011; Giammona and Craparo 2018; Singhvi et al. 2019; Su et al. 2020).

**Fig. 1:**
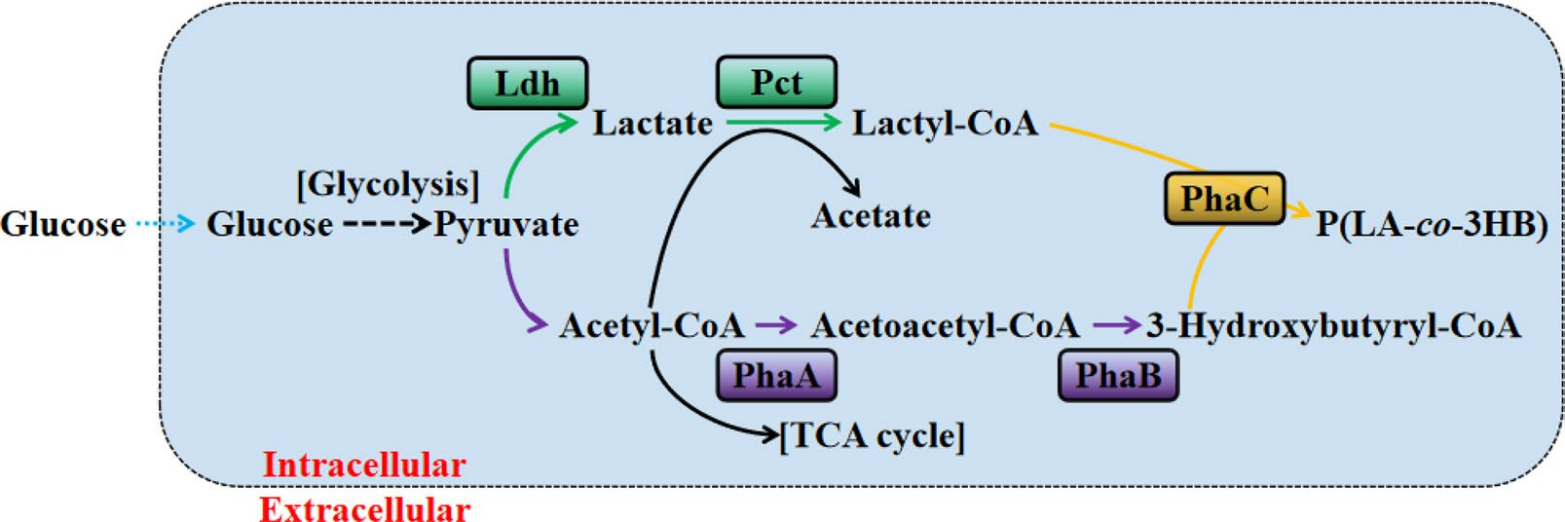
Synthetic pathway of P(LA-*co*-3HB) in recombinant *Escherichia coli*. Letters in boxes indicate enzymes. *Ldh* lactate dehydrogenase, *Pct* propionyl-CoA transferase, *PhaA* β-ketothiolase, *PhaB* NADPH-dependent acetoacetyl-CoA reductase, *PhaC* PHA synthase

In 2008, Taguchi et al. (2008) firstly established a one-step microbial metabolic process for the synthesis of a representative lactate-based copolymer, P(LA-*co*-3HB) (Fig. 1). The disadvantages of residues, which are generated in the process of chemical synthesis of the lactate-based copolymers, are effectively avoided by using the biosynthesis method. In addition, isolated microbial enzymes (such as lipase) also have been successfully used as catalysts for the production of the copolymers with diverse structures, various compositions and properties (Jiang and Zhang 2013).

In P(LA-*co*-3HB), the molecular weight of it will decrease with LA monomer incorporating into the copolymer (Yamada et al. 2011). Thermodynamic analysis reveals that melting (*T*_m_) and glass transition (*T*_g_) temperatures of the copolymer vary with the change of the mole percentage of LA monomer fraction. The copolymer with the higher mole percentage of LA monomer fraction usually has a lower melting temperature (*T*_m_) and a higher glass transition temperature (*T*_g_) (Yamada et al. 2009, 2010, 2011; Ishii et al. 2017). However, Yamada et al. (2010) point out that the change of glass transition temperature (*T*_g_) also needs to consider the molecular weight of the copolymer. In terms of the mechanical properties, Young’s modulus of the copolymer is lower than that of the homopolymer, and it decreases with the increase of the mole percentage of LA monomer fraction. The elongation at break of the copolymer is higher than that of the homopolymer and can be maintained for a relatively long time (Yamada et al. 2011; Ishii et al. 2017). The crystallinity of cast film [mainly due to the crystallization of 3-hydroxybutyrate (3HB)] decreases with the increase of LA monomer fraction (Yamada et al. 2010; Ishii et al. 2017). When the mole percentage of LA monomer fraction of the copolymer is higher than 15%, the transparency of the copolymer film increases significantly (Yamada et al. 2011).

It is meaningful to compare the material properties of P(LA-*co*-3HB) with the homopolymers PLA and poly(3-hydroxybutyrate) [P(3HB)]. PLA is a rigid, transparent, and compostable biodegradable material, P(3HB) is rigid and opaque but has high biodegradability, while P(LA-*co*-3HB) combines the advantages of PLA and P(3HB) in terms of transparency and biodegradability (Taguchi and Matsumoto 2020). Whereas, compared with PLA, PHAs containing 3HB, 3-hydroxyvalerate (3HV), and 4-hydroxybutyrate (4HB) have a higher hazard (cytotoxicity) due to their relatively low acidity and bioactivity (Singh et al. 2019). In addition, the hydrophobicity of the lactate-based copolymers lead to its poor biocompatibility, which will limit its application in some fields (such as medical field). These existing shortcomings of the lactate-based copolymers indicate that further studies and developments are needed before their commercialization. While the commercialization of the lactate-based copolymers has not been reported, in recent years there are more and more researches focus on P(LA-*co*-3HB). The properties [enantiomeric purity; sequential structure and molecular weight; thermal and mechanical properties (Nduko and Taguchi 2019)] of P(LA-*co*-3HB) are affected by the mole percentage of incorporated monomers, and different properties will affect its application in different fields. Therefore, it is very important to regulate the mole percentage of the monomer composition in the copolymers, especially to increase the mole percentage of LA monomer fractions in the copolymers. However, researchers will face the problems of the poor specificity of precursor supply enzymes and PHA synthases, as well as the inappropriate chassis cells, etc. So it is necessary to adjust microbial metabolic pathways through genetic engineering, fermentation engineering, etc. (Park et al. 2012a; Matsumoto and Taguchi 2013a, b).

Although the main contents of this article are the different production strategies of biosynthetic P(LA-*co*-3HB), the representative lactate-based copolymer, other microbial synthesis strategies of PHA containing LA are also introduced here (Table 1).

**Table 1:** Different production strategies for P(LA-*co*-3HB)

Authors	Strategies	Engineered microorganisms	Key characteristics	Main substrates	Lactate-based copolymer production statuses
Taguchi et al. (2008)	Enzyme engineering	*E. coli* JM109	Checking effect of PhaC from *Pseudomonas* sp. 61–3 with mutagenesis of two sites (S325T and Q481K); adding 10 mM calcium pantothenate to increase LA monomer fraction	20 g/L glucose	Engineered *E. coli* expressing PhaC1_*Ps*_(ST/QK) and Pct_*Me*_ can synthesize 19 wt% P(6 mol% LA-*co*-3HB); *M*_n_: (×10^5^) = 1.9, *M*_w_: (×10^5^) = 4.8, *M*_w_/*M*_n_ = 2.4
Yamada et al. (2010)		*E. coli* BW25113	Deleting *pflA*; checking effect of PhaC1_*Ps*_(ST/QK) with mutagenesis of F392; adding 10 mM calcium pantothenate	20 g/L glucose	*E. coli* JW0885 expressing PhaC1_*Ps*_(ST/FS/QK) and Pct_*Me*_ can synthesize 62 wt% P(45 mol% LA-*co*-3HB) with an increased PHA content and the highest LA monomer fraction; *M*_n_: (×10^4^) = 2, *M*_w_: (×10^4^) = 8, *M*_w_/*M*_n_ = 4.0
Yang et al. (2010)		*E. coli* XL1-Blue	Checking effect of the mutants of four sites in PhaC1_*Ps*6-19_ (E130, S325, S477, and Q481) and two mutants of Pct_*Cp*_ (Pct532_*Cp*_ and Pct540_*Cp*_)	20 g/L glucose	Engineered *E. coli* expressing different enzymes can synthesize different copolymers
Yang et al. (2011)		*E. coli* XL1-Blue	Checking effect of four kinds of engineering-type II PhaC1s with mutagenesis of four sites (E130, S325, S477, and Q481)	20 g/L glucose	Engineered *E. coli* expressing different PhaC1s and Pct540_*Cp*_ can synthesize different copolymers
Ochi et al. (2013)		*E. coli* LS5218	Introducing *phaJ4*; checking effect of PhaC from *C. necator* with mutagenesis of A510	5 g/L (*R*)-LA; 3 g/L sodium dodecanoate	Engineered *E. coli* expressing PhaC_*Re*_(AS) and Pct_*Me*_ can mainly synthesize P(5 mol% LA-*co*-3HB) with a relatively high PHA production and a stable LA monomer fraction; *M*_n_: (×10^5^) = 1.4, *M*_w_: (×10^5^) = 3.2, *M*_w_/*M*_n_ = 2.3
Kim et al. (2016)		*E. coli* XL1-Blue	Checking effect of Pct from *C. beijerinckii*, *C. perfringens*, and *K. pneumoniae*	20 g/L glucose	Engineered *E. coli* expressing PhaC1437_*Ps*6-19_ and Pct of *C. perfringens* can synthesize 10.6 wt% P(13.6 mol% LA-*co*-3HB)
David et al. (2017)		*E. coli* XL1-Blue	Checking effect of Bct from four strains from different genus	20 g/L glucose	Engineered *E. coli* expressing PhaC1437_*Ps*6-19_ and different Bcts can synthesize different copolymers
Yang et al. (2018)		*E. coli* XL1-Blue	Checking effect of FldA and HadA; introducing the aromatic copolymer production module	20 g/L glucose	Engineered *E. coli* expressing PhaC1437_*Ps*6-19_ can synthesize the copolymers containing d-phenyllactate and d-4-hydroxyphenyllactate
Jung et al. (2010)	Metabolic pathway construction	*E. coli* XL1-Blue	Deleting *ackA*, *ppc*, and *adhE*; replacing *ldhA* and *acs* natural promoters with *trc*; strains lacking *ppc* need additional 4 g/L sodium succinate	20 g/L glucose	*E. coli* JLX10 expressing PhaC1310_*Ps*6-19_ and Pct540_*Cp*_ can synthesize 19 wt% P(60 mol% LA-*co*-3HB); expressing PhaC1400_*Ps*6-19_ and Pct532_*Cp*_ can synthesize 46 wt% P(70 mol% LA-*co*-3HB)
Jung and Lee (2011)		*E. coli* XB-F	Deleting *lacI*, *pflB*, *frdABCD*, and *adhE*; replacing *ldhA* and *acs* natural promoters with *trc*; strains lacking *lacI* does not need additional IPTG	20 g/L glucose	*E. coli* JLXF5 expressing PhaC1310_*Ps*6-19_ and Pct540_*Cp*_ can synthesize 15.2 wt% P(67.4 mol% LA-*co*-3HB)
Nduko et al. (2014)		*E. coli* BW25113	Deleting *pflA*, *pta*, *ackA*, *poxB*, and *dld*; overexpressing GatC to increase the utilization of xylose; adding 10 mM calcium pantothenate	20/30/40 g/L xylose	*E. coli* JWMB1 expressing PhaC1_*Ps*_(ST/FS/QK) and Pct_*Me*_ can synthesize 58 wt% P(73 mol% LA-*co*-3HB) from 20 g/L xylose; *M*_n_: (×10^4^) = 1.7, *M*_w_: (×10^4^) = 3.8, *M*_w_/*M*_n_ = 2.2
Salamanca-Cardona et al. (2014a)		*E. coli* LS5218	Deleting *pflA*	20 g/L xylose	*E. coli* RSC10 expressing PhaC1_*Ps*_(ST/QK) and Pct_*Me*_ can synthesize 41.1 wt% P(8.3 mol% LA-*co*-3HB)
Kadoya et al. (2015a)		*E. coli* BW25113	Deleting RpoS, RpoN, FliA, and FecI, which are *E. coli* non-essential σ factors; adding 10 mM calcium pantothenate	20 g/L glucose	*E. coli* JW3169 expressing PhaC1_*Ps*_(ST/QK) and Pct_*Me*_ can synthesize 75.1 wt% P(26.2 mol% LA-*co*-3HB)
Kadoya et al. (2015a)		*E. coli* BW25113	Deleting *mtgA*, which plays an auxiliary role in peptidoglycan synthesis; adding 10 mM calcium pantothenate	20 g/L glucose	*E. coli* JW3175 expressing PhaC1_*Ps*_(ST/QK) and Pct_*Me*_ can synthesize 7.0 g/L copolymer with an increased yield
Kadoya et al. (2017)		*E. coli* BW25113	Deleting PdhR, CspG, YneJ, ChbR, YiaU, CreB, YgfI, and NanK, which are *E. coli* non-lethal transcription factors; adding 10 mM calcium pantothenate	30 g/L glucose	Engineered *E. coli* expressing PhaC1_*Ps*_(ST/QK) and Pct_*Me*_ with an increased copolymer yield (6.2–10.1 g/L)
Kadoya et al. (2018)		*E. coli* BW25113	Overexpression Mlc, which is a multiple regulator of glucose and xylose uptake; adding 10 mM calcium pantothenate	50 g/L mixed sugar (wt%, glucose:xylose = 4:1)	Engineered *E. coli* expressing PhaC1_*Ps*_(ST/QK) and Pct_*Me*_ can synthesize 64.9 wt% P(11.8 mol% LA-*co*-3HB); *M*_n_: (×10^4^) = 6.7, *M*_w_: (×10^4^) = 9.4, *M*_w_/*M*_n_ = 1.4
Yang et al. (2018)		*E. coli* XL1-Blue	Deleting *ldhA*, *adhE*, *pflB*, *frdB*, and *poxB*; introducing the aromatic copolymer production module	20 g/L glucose	Engineered *E. coli* expressing PhaC1437_*Ps*6-19_ can synthesize the copolymers containing d-phenyllactate and d-4-hydroxyphenyllactate
Goto et al. (2019b)		*E. coli* DH5*α* *E. coli* LS5218 *E. coli* XL1-Blue	Introducing *ldhD*	20 g/L glucose	Engineered *E. coli* expressing PhaC1_*Ps*_(ST/QK) and Pct_*Me*_ can synthesize the copolymers with an increased LA monomer fraction compared with original strains
Lu et al. (2019)		*E. coli* MG1655	Deleting *ubiX* to attenuate respiratory chain; deleting *dld*; checking effect of different concentrations of IPTG and l-arabinose	20 g/L glucose	*E. coli* JX041 expressing PhaCm(ED/ST/QK) and Pct540_*Cp*_ can synthesize 81.7 wt% P(14.1 mol% LA-*co*-3HB)
Wei et al. (2021)		*E. coli* MG1655	Deleting *ydiI*, *yciA*, and *dld*	20 g/L glucose; 20 g/L xylose	*E. coli* WXJ03 expressing PhaCm(ED/ST/QK) and Pct540_*Cp*_ can synthesize 66.3 wt% P(46.1 mol% LA-*co*-3HB) from xylose
Wu et al. (2021)		*E. coli* MG1655	Deleting *ubiX*, *ptsG*, and *dld*	10 g/L mixed sugar (wt%, glucose:xylose = 7:3)	*E. coli* WJ03 expressing PhaCm(ED/ST/QK) and Pct540_*Cp*_ can synthesize P(7 mol% LA-*co*-3HB)
Nduko et al. (2013)	Different substrates	*E. coli* BW25113	Deleting *pflA*; checking effect of glucose and xylose; adding 10 mM calcium pantothenate	20 g/L glucose; 20 g/L xylose	*E. coli* JW0885 expressing Pct_*Me*_ produces the copolymer have a higher LA monomer fraction (34 mol%) from xylose; *M*_n_: (×10^4^) = 4.0, *M*_w_: (×10^4^) = 17, *M*_w_/*M*_n_ = 4.1; PhaC1_*Ps*_(ST/FS/QK) has a better effect than PhaC1_*Ps*_(ST/QK)
Oh et al. (2014)		*E. coli* W	Sucrose is broken down into fructose and glucose in vivo	20 g/L sucrose	Engineered *E. coli* expressing PhaC1437_*Ps*6-19_ and Pct540_*Cp*_ can synthesize 12.2 wt% P(16 mol% LA-*co*-3HB); *M*_n_: (×10^4^) = 1.53, *M*_w_: (×10^4^) = 2.78, *M*_w_/*M*_n_ = 1.82
Salamanca-Cardona et al. (2014b)		*E. coli* LS5218	Introducing *xylB* and *xynB*; adding single sugar to assist the copolymer production from beechwood xylan	10 g/L xylan; combination of a single sugar substrate (20 g/L xylose or arabinose) and xylan (10 g/L)	Engineered *E. coli* expressing PhaC1_*Ps*_(ST/QK) and Pct_*Me*_ can synthesize 40.4 wt% P(2.1 mol% LA-*co*-3HB) from xylose and xylan; *M*_n_: (×10^5^) = 1.18, *M*_w_: (×10^5^) = 2.78, *M*_w_/*M*_n_ = 2.35; 30.3 wt% P(3.7 mol% LA-*co*-3HB) from arabinose and xylan; *M*_n_: (×10^5^) = 1.13, *M*_w_: (×10^5^) = 3.03, *M*_w_/*M*_n_ = 2.67
Salamanca-Cardona et al. (2014a)		*E. coli* LS5218	Deleting *pflA*; using xylose and acetate to simulate xylan derived from beechwood	20 g/L xylose; 33 mM acetate	*E. coli* RSC10 expressing PhaC1_*Ps*_(ST/QK) and Pct_*Me*_ can synthesize 41.1 wt% P(8.3 mol% LA-*co*-3HB) from xylose; 4.2 wt% P(18.5 mol% LA-*co*-3HB) from xylose and acetate; adding acetate increases LA monomer fraction
Oh et al. (2015)		*E. coli* XL1-Blue *C. necator*	Introducing *ldhA* into *C. necator*; rice bran hydrolysates are purified by a series of processes and concentrated; resulting solution contains 16.3% (w/w) glucose	10 mL/L rice bran hydrolysate solution (corresponds to 20 g/L glucose and 3.4 g/L fructose)	Engineered *E. coli* expressing PhaC1437_*Ps*6-19_ and Pct540_*Cp*_ can synthesize 82.3 wt% P(28.6 mol% LA-*co*-3HB); *C. necator* 437–540 expressing the same enzymes can synthesize 35.8 wt% P(7.3 mol% LA-*co*-3HB)
Sun et al. (2016)		*E. coli* BW25113	Lignocellulosic biomass is treated by NaClO_2_ and NaOH in the two-step process to obtain the highest total sugar yield; the two-step process does not produce the toxic hydrolysates; the hydrolysates mainly include glucose, xylose, and trace arabinose; adding 10 mM calcium pantothenate	*Miscanthus* × *giganteus* (hybrid *Miscanthus*); rice straw hydrolysate solution	Engineered *E. coli* expressing PhaC1_*Ps*_(ST/QK) and Pct_*Me*_ basically has no effect on content, yield, and LA monomer fraction of the copolymer when using the hydrolysate solution derived from hybrid *Miscanthus*; *M*_n_: (×10^4^) = 7.7 *M*_w_: (×10^4^) = 37.0, *M*_w_/*M*_n_ = 4.8; decreases LA monomer fraction when using the hydrolysate solution derived from rice straw; *M*_n_: (×10^4^) = 7.1, *M*_w_: (×10^4^) = 36.2, *M*_w_/*M*_n_ = 5.1
Salamanca-Cardona et al. (2017)		*E. coli* BW25113 *E. coli* BW25113 (Δ*pflA*) *E. coli* LS5218 *E. coli* LS5218 (Δ*pflA*)	*E. coli* LS5218 (Δ*pflA*) is an acetate-tolerant strain and even produces the copolymer using acetate as a sole carbon source	20 g/L xylose; 25 mM acetate	*E. coli* RSC10 expressing PhaC1_*Ps*_(ST/QK) and Pct_*Me*_ can synthesize 45.3 wt% P(13.1 mol% LA-*co*-3HB); *M*_n_: (×10^4^) = 6.3, *M*_w_: (×10^4^) = 26.5, *M*_w_/*M*_n_ = 5.0
Takisawa et al. (2017)		*E. coli* BW25113	Woody extract is treated by a series of processes and the hemicellulosic hydrolysate’s total sugar concentration is 154.5 g/L; the purified hydrolysates’ total sugar concentration are 133.0 and 62.4 g/L for active charcoal treatment and ion-exchange resin treatment, respectively; adding 10 mM calcium pantothenate	The hemicellulosic hydrolysate solution derived from dissolving pulp manufacturing-obtained woody extract; 0/1/2/5/10 g/L acetate	Engineered *E. coli* expressing PhaC1_*Ps*_(ST/QK) and Pct_*Me*_ can synthesize 62.4 wt% P(5.5 mol% LA-*co*-3HB); *M*_n_: (×10^4^) = 6.9, *M*_w_: (×10^4^) = 48, *M*_w_/*M*_n_ = 7.0; adding acetate decreases PHA content and LA monomer fraction
Kadoya et al. (2018)		*E. coli* BW25113	Hybrid *Miscanthus* is treated by NaClO_2_ and NaOH in the two-step process; assuming a small amount of acetate in the hydrolysate inhibits the copolymerization of LA; adding 10 mM calcium pantothenate	*Miscanthus* × *giganteus* (hybrid *Miscanthus*) hydrolysate solution	Engineered *E. coli* expressing PhaC1_*Ps*_(ST/QK) and Pct_*Me*_ decreases LA monomer fraction; *M*_n_: (×10^4^) = 6.7, *M*_w_: (×10^4^) = 9.4, *M*_w_/*M*_n_ = 1.4
Sohn et al. (2020)		*E. coli* DH5*α* *E. coli* JM109 *E. coli* Top10 *E. coli* W3110 (Δ*lacI*) *E. coli* XL1-Blue *E. coli* XL10-Gold	Introducing *sacC*; sucrose is broken down into fructose and glucose; all *E. coli* produces the copolymers	20 g/L sucrose	Engineered *E. coli* XL1-Blue expressing PhaC1437_*Ps*6-19_ and Pct540_*Cp*_ can synthesize P(42.3 mol% LA-*co*-3HB) with the highest concentration (0.576 g/L) and a relatively high content (29.44 wt%)
Wu et al. (2021)		*E. coli* MG1655	Deleting *ubiX*, *ptsG*, and *dld*; corn straw is hydrolyzed with sodium hydroxide solution and the impurities are removed by active charcoal treatment and filtration	Corn straw hydrolysate solution	*E. coli* WJ03 expressing PhaCm(ED/ST/QK) and Pct540_*Cp*_ can synthesize P(7.1 mol% LA-*co*-3HB)
Yamada et al. (2009)	Culture conditions	*E. coli* W3110	Culturing in anaerobic conditions after 24 h aerobic cultivation; adding 10 mM calcium pantothenate	20 g/L glucose	Engineered *E. coli* expressing PhaC1_*Ps*_(ST/QK) and Pct_*Me*_ can synthesize 2 wt% P(47 mol% LA-*co*-3HB); *M*_n_: (×10^4^) = 1.5, *M*_w_: (×10^4^) = 2.0, *M*_w_/*M*_n_ = 1.3
Yamada et al. (2010)		*E. coli* BW25113	Deleting *pflA*; culturing in anaerobic conditions after 16 h aerobic cultivation in a jar fermentor; adding 10 mM calcium pantothenate	20 g/L glucose	*E. coli* JW0885 expressing PhaC1_*Ps*_(ST/QK) and Pct_*Me*_ can synthesize 15 wt% P(47 mol% LA-*co*-3HB); *M*_n_: (×10^4^) = 2, *M*_w_: (×10^4^) = 6, *M*_w_/*M*_n_ = 3.0; expressing PhaC1_*Ps*_(ST/FS/QK) and Pct_*Me*_ can synthesize 12 wt% P(62 mol% LA-*co*-3HB); *M*_n_: (×10^4^) = 1, *M*_w_: (×10^4^) = 4, *M*_w_/*M*_n_ = 4.0; anaerobic conditions decrease the copolymer content and increase LA monomer fraction
Yang et al. (2010)		*E. coli* XL1-Blue	Adjusting DOC (30%, 10%), pH (by 28% (v/v) ammonia water) and glucose concentration in a jar fermentor	15/5 g/L glucose	Engineered *E. coli* expressing PhaC1310_*Ps*6-19_ and Pct540_*Cp*_ can adjust LA monomer fraction ranging from 8.7 to 64.4 mol%
Jung and Lee (2011)		*E. coli* XB-F	Deleting *lacI*, *pflB*, *frdABCD*, and *adhE*; replacing *ldhA* and *acs* natural promoters with *trc*; strains lacking *lacI* does not need additional IPTG; adjusting DOC (above 40%), pH (by 28% (v/v) ammonia water) in a jar fermentor	20 g/L glucose	*E. coli* JLXF5 expressing PhaC1310_*Ps*6-19_ and Pct540_*Cp*_ can synthesize 43 wt% P(39.6 mol% LA-*co*-3HB) in about 80 h; molecular weight: (×10^5^) = 1.41
Yamada et al. (2011)		*E. coli* JM109 *E. coli* BW25113 (Δ*pflA*)	Culturing in anaerobic conditions after 16 h aerobic cultivation in a jar fermentor; adding 10 mM calcium pantothenate	20 g/L glucose	*E. coli* JW0885 expressing PhaC1_*Ps*_(ST/QK) and Pct_*Me*_ can adjust LA monomer fraction ranging from 29 to 47 mol%
Oh et al. (2015)		*E. coli* XL1-Blue *C. necator*	Introducing *ldhA* into *C. necator*; adjusting pH by 28% (v/v) NH_4_OH in a jar fermentor; the utilization of batch fermentation overcomes the bottleneck of the utilization of fructose	100 mL/L rice bran hydrolysate solution	Engineered *E. coli* expressing PhaC1437_*Ps*6-19_ and Pct540_*Cp*_ can synthesize 53.89 wt% P(3.63 mol% LA-*co*-3HB) in 39 h resulting in the highest LA monomer fraction; *C. necator* 437–540 expressing the same enzymes can’t use up fructose in 63 h; *M*_n_: (×10^4^) = 2.19, *M*_w_: (×10^4^) = 4.16, *M*_w_/*M*_n_ = 1.90
David et al. (2017)		*E. coli* XL1-Blue	Adjusting pH by 28% (v/v) NH_4_OH in a jar fermentor	20 g/L glucose	Compared with Pct540_*Cp*_, engineered *E. coli* expressing PhaC1437_*Ps*6-19_ and Bct_*Eh*_ shows higher OD_600_ and weight percentage, but LA monomer fraction is lower
Yang et al. (2018)		*E. coli* XL1-Blue	Introducing the aromatic copolymer production module; using fed-batch fermentation, which is performed by the pH-stat strategy and the pulsed-feeding strategy; adjusting DOC (above 40%) and pH (by 28% (v/v) ammonia solution) in a jar fermentor	20/10 g/L glucose	Engineered *E. coli* expressing PhaC1437_*Ps*6-19_ can synthesize aromatic PHAs to a reasonably high concentration
Goto et al. (2019b)		*E. coli* DH5*α* *E. coli* LS5218 *E. coli* XL1-Blue	Introducing *ldhD*; creating relatively anaerobic conditions (shaking speed = 0/60 strokes/min)	20 g/L glucose	Engineered *E. coli* expressing PhaC1_*Ps*_(ST/QK) and Pct_*Me*_ can synthesize the copolymers with an increased LA monomer fraction in relatively anaerobic conditions
Hori et al. (2019)		*E. coli* MG1655	Initial sugar is glucose; feed is xylose, and its concentration rises with time; adjusting pH by 4 N NaOH in a jar fermentor	20 g/L glucose; 5/10/30 g/L xylose	Engineered *E. coli* expressing PhaC1_*Ps*_(ST/QK) and Pct_*Me*_ can synthesize 44.3 wt% P(4.9 mol% LA-*co*-3HB); *M*_n_: (×10^4^) = 2.8, *M*_w_: (×10^4^) = 16, *M*_w_/*M*_n_ = 5.7
Sohn et al. (2020)		*E. coli* XL1-Blue	Introducing *sacC*; adjusting pH by 28% (v/v) NH_4_OH in a jar fermentor; the utilization of batch fermentation overcomes the bottleneck of the utilization of fructose	20 g/L sucrose	Engineered *E. coli* expressing PhaC1437_*Ps*6-19_ and Pct540_*Cp*_ can synthesize 20.88 wt% P(38 mol% LA-*co*-3HB) in 28 h
Song et al. (2012)	Non-traditional chassis cells	*C. glutamicum*	Introducing *ldhA*; adding 0.45 mg/L biotin causes *C. glutamicum* not to produce glutamate	60 g/L glucose	Engineered *C. glutamicum* expressing PhaC1_*Ps*_(ST/QK) and Pct_*Me*_ can synthesize 2.4 wt% P(96.8 mol% LA-*co*-3HB); *M*_n_: (×10^3^) = 5.2, *M*_w_: (×10^3^) = 7.4, *M*_w_/*M*_n_ = 1.4
Park et al. (2013b)		*C. necator*	Introducing *ldhA*	20 g/L glucose	*C. necator* 437–540 expressing PhaC1437_*Ps*6-19_ and Pct540_*Cp*_ can synthesize 33.9 wt% P(37 mol% LA-*co*-3HB)
Park et al. (2015)		*C. necator*	Introducing *sacC* and *ldhA*; adjusting pH by 28% (v/v) NH_4_OH in a jar fermentor	20 g/L sucrose	*C. necator* 437–540 expressing PhaC1437_*Ps*6-19_ and Pct540_*Cp*_ can synthesize 19.5 wt% P(21.5 mol% LA-*co*-3HB); *M*_n_: (×10^4^) = 2.19, *M*_w_: (×10^4^) = 4.17, *M*_w_/*M*_n_ = 1.90
Tran and Charles (2016)		*S. meliloti* Rm1021	Replacing *phbC* with PhaC1400_*Ps*6-19_ and Pct532_*Cp*_	Yeast mannitol	*S. meliloti* SmUW254 expressing PhaC1400_*Ps*6-19_ and Pct532_*Cp*_ can synthesize P(30 mol% LA-*co*-3HB)

Enzymes related to copolymer synthesis which are not specifically described include propionyl-CoA transferase (Pct), β-ketothiolase (PhaA), and NADPH-dependent acetoacetyl-CoA reductase (PhaB). If engineered strains do not have a 3HB synthesis pathway, 3HB needs to be added to the medium

*M*_n_: number-average molecular weight; *M*_w_: weight-average molecular weight; *M*_w_/*M*_n_: polydispersity index

## Different engineered strategies of *E. coli* for P(LA-*co*-3HB) biosynthesis

### Enzyme engineering

The wild-type *E. coli* cannot provide D-LA-CoA, which is required for copolymer synthesis, so CoA transferase is needed to produce it. Propionate-CoA transferase of *Clostridium propionicum* (Pct_*Cp*_) is one of the most representative CoA transferases. In addition, Pct of *Megasphaera elsdenii* has also been used, which contribute to the heterologous expression in *E. coli* (Taguchi et al. 2008). Different precursors containing CoA require polymerization of PhaC, and the PhaC that can polymerize LA into the copolymers effectively is called LA-CoA polymerizing enzyme (LPE); the discovery of LPE is a decisive breakthrough in the biosynthesis of the lactate-based copolymers (Taguchi et al. 2008; Matsumoto and Taguchi 2013b). To adjust the monomer composition of the copolymers, researchers are committed to discovering new enzymes or improving the activity and the substrate specificity of existing enzymes by random mutagenesis, screening, or structure prediction based on homologous sequences of identified enzymes (Choi et al. 2020b).

LPE is created by introducing double mutations, S325T and Q481K, into PHA synthase 1 (PhaC1_*Ps*6-19_) of *Pseudomonas* sp. 61-3 (Taguchi et al. 2008; Tajima et al. 2009). To find the same effect, the same mutations are introduced into *Pseudomonas* sp. MBEL 6-19 at the corresponding sites of PHA synthase 1 (PhaC1_*Ps*6-19_). The mutant enzyme cannot polymerize LA-CoA into the copolymers effectively. Its activity can be improved by gene mutations of its four sites (E130, S325, S477, and Q481), which are previously addressed through evolutionary engineering studies performed by Taguchi’s group (Taguchi and Doi 2004; Shozui et al. 2009) (Yang et al. 2010). Further engineered type II *Pseudomonas* PHA synthases 1 (PhaC1s) are obtained from *Pseudomonas chlororaphis*, *Pseudomonas* sp. 61-3, *Pseudomonas putida* KT2440, *Pseudomonas resinovorans*, and *Pseudomonas aeruginosa* PAO1 by mutagenesis of four sites (E130, S325, S477, and Q481) (Yang et al. 2011). Base on the PhaC1_*Ps*6-19_ (Taguchi et al. 2008), another mutant, F392, is obtained. Engineered *E. coli* BW25113 (with mutated PHA synthase, F392S) along with the pyruvate formate lyase activating enzyme gene (*pflA*) deletion can synthesize 62 wt% P(45 mol% LA-*co*-3HB) in a medium containing 20 g/L glucose with the highest LA monomer fraction (Yamada et al. 2010). Ren et al. (2017) examine the mutation effects of PhaC1 (E130D, S325T, F392S, S477G, and Q481K) and PhaC2 (S326T, S478G, and Q482K) of *Pseudomonas stutzeri*. Lu et al. (2019) examine the mutation effects of PhaCm (E130D, S325T, and Q481K) of *Pseudomonas fluorescens*. In addition, it should be noted that LPE has a strict substrate specificity toward D-LA-CoA, which is obtained from enantiomer analysis of P(LA-*co*-3HB) synthesized in vivo and analysis of LPE in vitro, the synthesized copolymers by LPE are almost entirely composed of D-LA (Tajima et al. 2009; Yamada et al. 2009; Matsumoto and Taguchi 2013b).

Type I PHA synthase of *Cupriavidus necator* (PhaC_*Re*_) exhibits activity toward 2-hydroxybutyryl-CoA (2HB-CoA) in vitro (Han et al. 2011). Similar to position 481 in type II PHA synthase (PhaC1_*Ps*6-19_) (Taguchi et al. 2008), PhaC_*Re*_ is mutated at position 510. Partially engineered *E. coli* LS5218 (A510X) can synthesize the copolymers in a medium with 5 g/L (*R*)-LA and 3 g/L sodium dodecanoate, indicating that 510 residue plays a key role in LA polymerization (Ochi et al. 2013).

Pct_*Cp*_ cannot convert LA into LA-CoA effectively, and it also exert the inhibitory effects on cell growth (Yang et al. 2010). While when some sites of Pct_*Cp*_ are mutated, its activity can be promoted and the inhibition of cell growth can be alleviated. Two beneficial Pct_*Cp*_ mutants have been constructed to achieve these two goals. One mutant is Pct532_*Cp*_, within which with amino acid mutation of A243T and A1200G (silent nucleotide mutation). Another mutant is Pct540_*Cp*_ with amino acid mutation of V193A and four silent nucleotide mutations of T78C, T669C, A1125G, as well as T1158C (Yang et al. 2010). Engineered *E. coli* XL1-Blue with the expression of the *phaC1437* gene of *Pseudomonas* sp. MBEL 6-19 and the *CB3819* gene (or the *CB4543* gene) of *Clostridium beijerinckii* can synthesize P(3HB) within a medium containing 20 g/L glucose and 2 g/L sodium 3HB. Engineered *E. coli* XL1-Blue with the expression of the *pct* gene of *Clostridium perfringens* can synthesize 10.6 wt% P(13.6 mol% LA-*co*-3HB) in the same culture media too (Kim et al. 2016). Four butyryl-CoA transferases (Bct) of *Roseburia* sp., *Eubacterium hallii*, *Faecalibacterium prausnitzii*, and *Anaerostipes caccae* can polymerize LA, 2HB, and 3HB with different activities (David et al. 2017).

Moreover, cinnamoyl-CoA:phenyllactate CoA-transferase (FldA) of *Clostridium sporogenes* can transfer CoA from cinnamoyl-CoA to phenyllactate and 4-hydroxyphenyllactate; isocaprenoyl-CoA:2-hydroxyisocaproate CoA-transferase (HadA) of *Clostridium difficile* which can use acetyl-CoA as a CoA donor is further identified, and have been revealed that it has a wider substrate spectrum than FldA (Yang et al. 2018).

### Metabolic pathway engineering

Lactyl-CoA and acetyl-CoA are two precursors of P(LA-*co*-3HB), both of them are derived from pyruvate. These indicate that regulation of metabolic flux of pyruvate is an effective method to adjust LA monomer fraction in the copolymers. The overexpression of the key pathway genes or blocking competitive pathways are both effective metabolic engineering strategies to achieve this gold. Except for these two strategies, there are still some other methods that can adjust LA monomer fraction in the copolymers.

The deletion of the acetate kinase gene (*ackA*) and the phosphoenolpyruvate carboxylase gene (*ppc*), as well as the replacement of the native promoter of the d-lactate dehydrogenase gene (*ldhA*) with the *trc* promoter, can regulate the metabolic flux by rational engineering. In addition, the deletion of the acetaldehyde/alcohol dehydrogenase gene (*adhE*) and the replacement of the native promoter of the acetyl-CoA synthetase gene (*acs*) with the *trc* promoter by in silico gene knockout simulation as well as flux response analysis can further regulate the metabolic flux. Engineered *E. coli* XL1-Blue increased the copolymer content and LA monomer fraction up to ca. 3.7-fold (in case of expressing PhaC1310_*Ps*6-19_, Pct540_*Cp*_, and PhaAB_*Cn*_) and 2.6-fold (in case of expressing PhaC1400_*Ps*6-19_, Pct532_*Cp*_, and PhaAB_*Cn*_), respectively (Jung et al. 2010). With the deletion of the pyruvate formate lyase gene (*pflB*), the fumarate reductase gene (*frdABCD*), and the *adhE* gene, engineered *E. coli* XB-F can synthesize 15.2 wt% P(67.4 mol% LA-*co*-3HB) in a medium containing 20 g/L glucose along with the *trc* promoter replacement of the *ldhA* and *acs* genes (Jung and Lee 2011). Partial deletion of other target genes, such as the *pflA* gene, the phosphate acetyltransferase gene (*pta*), the pyruvate oxidase gene (*poxB*), the NAD^+^-independent lactate dehydrogenase gene (*dld*), and the *ackA* gene, are also conducive to improve the copolymers. With the deletion of the *pflA* and *dld* genes, engineered *E. coli* BW25113 can synthesize 58 wt% P(73 mol% LA-*co*-3HB) in a medium containing 20 g/L xylose. In addition, the overexpression of non-ATP consuming galactitol permease (GatC) to promote the absorption of xylose can increase the copolymer yield and LA monomer fraction of some mutants (Nduko et al. 2014). PflB is regulated by PflA, then the deletion of the *pflA* gene can increase the flux of pyruvate into LA and acetyl-CoA. Engineered *E. coli* LS5218 can synthesize 45.1 wt% P(0.9 mol% LA-*co*-3HB) in a medium with 20 g/L glucose and 41.1 wt% P(8.3 mol% LA-*co*-3HB) in a medium with 20 g/L xylose, respectively. However, when compared with the wild-type strain, the increase of LA monomer fraction could be offset for the significantly lower total cell biomass (Salamanca-Cardona et al. 2014a). Engineered *E. coli* BW25113 along with the deletion of the monofunctional peptidoglycan transglycosylase gene (*mtgA*) can enhance the copolymer production. Simultaneously, with the deletion of *mtgA*, the widths of the mutant cells become wilder than that of the wild-type cells (Kadoya et al. 2015a). The introduction of the D-LDH gene (*ldhD*) of *Lactobacillus acetotolerans* HT into different *E. coli* allows the improvement of LA monomer fraction (Goto et al. 2019b). Moreover, one-step production of the copolymers containing phenyllactate and 4-hydroxyphenyllactate from glucose by engineered *E. coli* XL1-Blue is designed. The globally metabolic engineering strategy of this one-step production of the copolymers includes the deletions of the *ldhA*, *adhE*, *pflB*, *frdB*, and *poxB* genes (Yang et al. 2018).

The cultivation of *E. coli* with mixed sugar will cause carbon catabolite repression, which can be derepressed by the overexpression of Mlc, a multiple regulator of glucose and xylose uptake. Engineered *E. coli* BW25113 can synthesize 64.9 wt% P(11.8 mol% LA-*co*-3HB) in a medium with 50 g/L mixed sugar (wt%, glucose:xylose = 4:1). In the same experiment, an increase in cell length is also observed, which is helpful for the accumulation of the copolymer (Kadoya et al. 2018).

Aim to change the copolymer production and the monomer composition, we can also disrupt σ factors, which globally govern the transcription of the corresponding genes. *E. coli* possesses four non-essential σ factors, RpoS, RpoN, FliA, and FecI. Engineered *E. coli* BW25113 along with the *rpoN* gene deletion can synthesize 75.1 wt% P(26.2 mol% LA-*co*-3HB) in a medium containing 20 g/L glucose, which is superior to that of the wild-type strain (Kadoya et al. 2015b). Furthermore, all of the deletions of non-lethal transcription factors of *E. coli* are screened by Keio Collection test. Among 252 mutants, eight of them, Δ*pdhR*, Δ*cspG*, Δ*yneJ*, Δ*chbR*, Δ*yiaU*, Δ*creB*, Δ*ygfI*, and Δ*nanK*, increase the copolymer yield (6.2–10.1 g/L) when compared to *E. coli* BW25113 (5.1 g/L) in a medium containing 30 g/L glucose with an insignificant change in cell density (Kadoya et al. 2017).

Attenuating respiratory chain increases the accumulation of LA in *E. coli* under aerobic conditions. The deletion of the flavin prenyltransferase gene (*ubiX*) can implement this strategy by attenuating the synthesis of coenzyme Q8, a key ingredient involved in respiratory chain in *E. coli*. Engineered *E. coli* MG1655 along with the *dld* gene deletion can synthesize 81.7 wt% P(14.1 mol% LA-*co*-3HB) in a medium containing 20 g/L glucose (Lu et al. 2019). On this basis, the Pct540_*Cp*_ promoter is replaced with the *ldhA* promoter, and the glucose-specific PTS enzyme IIBC component gene (*ptsG*) is knocked out to weaken carbon catabolite repression. Engineered *E. coli* MG1655 can synthesize P(7 mol% LA-*co*-3HB) in a medium containing 10 g/L mixed sugar (wt%, glucose:xylose = 7:3), but LA monomer fraction is decreased compared with strain without the *ptsG* gene deletion (Wu et al. 2021). Another strategy is proposed by the same research group too, which is aimed to delete the thioesterase genes (*ydiI* and *yciA*) to prevent the degradation of intracellular LA-CoA. Engineered *E. coli* MG1655 along with the *dld* gene deletion can synthesize 66.3 wt% P(46.1 mol% LA-*co*-3HB) in a medium containing 20 g/L xylose. It should be pointed out that the lack of thioesterase plays a major regulatory role (Wei et al. 2021). The presence of LA-CoA degrading enzymes (LDEs) (such as thioesterase) may lead to extremely low intracellular LA-CoA content in *E. coli*, which accounts for the efficient copolymer production (Matsumoto et al. 2018). The deletion of similar enzymes may increase LA monomer fraction in the copolymers while preventing the extension of the copolymer chain (Matsumoto et al. 2018). Potential LDEs [such as possible short-chain fatty acyl-CoA degrading enzymes (Clomburg et al. 2012)] are promising as elements for regulating the copolymer composition.

## Different culture conditions of *E. coli* for P(LA-*co*-3HB) biosynthesis

Introducing LPE and monomer synthesis enzymes into the *pflA* gene deleted *E. coli* BW25113, the copolymer in the mutant growing on 20 g/L xylose has a higher LA monomer fraction (34 mol%) than that growing on 20 g/L glucose (LA monomer fraction is 26 mol%). The utilization of evolved LPE (ST/FS/QK) can further enhance this advantage (Nduko et al. 2013). Introduction of the endoxylanase gene (*xylB*) of *Streptomyces coelicolor* and the *β*-xylosidase gene (*xynB*) of *Bacillus subtilis* into *E. coli* LS5218 allows converting xylan into PHA in vivo. Furthermore, when xylose or arabinose is added to the media at the same time, the production yields of PHA in engineered *E. coli* can increase up to 18-fold (Salamanca-Cardona et al. 2014b). Sucrose is undoubtedly one of the most abundant and the least expensive carbon sources. Engineered *E. coli* W can break down 20 g/L sucrose into fructose and glucose, and further to synthesize 12.2 wt% P(16 mol% LA-*co*-3HB) in vivo (Oh et al. 2014). To establish an efficient sucrose utilization pathway, the β-fructofuranosidase gene (*sacC*) of *Mannheimia succiniciproducens* MBEL55E is introduced into different genetically modified *E. coli* strains. Among the tested recombinant *E. coli* strains, engineered *E. coli* XL1-Blue synthesize the P(42.3 mol% LA-*co*-3HB) with the highest concentration of 0.576 g/L and a relatively high content of 29.44 wt% in a medium containing 20 g/L sucrose (Sohn et al. 2020).

Using traditional carbon sources such as glucose to produce the copolymers is a simple and effective method, but the copolymers’ increase in the production and the expansion of the use scope are inhibited by high raw material costs, so it is necessary to focus on the development of the inexpensive materials. By-products from different processing industries have great potentials, such as the residues of the biodiesel industry (Plácido and Capareda 2016), chitin and chitosan extracted from the marine waste resources (Yadav et al. 2019), the wastes of milk processing and reducing such as cheese whey (Zikmanis et al. 2020), lignocellulosic biomass and other green wastes (Langsdorf et al. 2021), as well as pulp and paper mill wastes (Haile et al. 2021). Some non-traditional carbon sources are not only beneficial to the copolymer production to a certain extent, but also can reduce the risk of the environmental pollution.

Rice bran is a by-product of the rice manufacturing process, and possesses certain potential as a feedstock for bio-based polymers. A rice bran treatment process has been developed to produce 43.7 kg hydrolysate solution containing 24.41 g/L glucose and a small amount of fructose from 5 kg rice bran (Oh et al. 2015). With the expression of LPE and monomer supplying enzymes, engineered *E. coli* XL1-Blue can synthesize 82.3 wt% P(28.6 mol% LA-*co*-3HB) in 10 mL/L hydrolysate solution; *C. necator* 437–540 can synthesize 35.8 wt% P(7.3 mol% LA-*co*-3HB) in the same condition (Oh et al. 2015).

P(LA-*co*-3HB) can be produced from glucose or xylose, which demonstrates the feasibility of using lignocellulosic-like biomass as a carbon source to produce P(LA-*co*-3HB). Wu et al. (2021) use corn stover hydrolysate to synthesize P(7.1 mol% LA-*co*-3HB) successfully, although cell growth is slightly inhibited. Compared with pure sugars, Sun et al. (2016) find that the hydrolysate solution derived from *Miscanthus* × *giganteus* (hybrid *Miscanthus*) does not affect content, yield, and LA monomer fraction of the copolymer, while the hydrolysate solution derived from rice straw decrease LA monomer fraction. However, some other researchers find that using the hydrolysate solution derived from hybrid *Miscanthus* will lead to a decrease in LA monomer fraction of the copolymer, which is suspected to be the effect of a small amount of acetate in the biomass sugar solution (Kadoya et al. 2018).

The hemicellulosic hydrolysate solution derived from dissolving pulp manufacturing-obtained woody extract is mainly composed of xylose and galactose, but a small amount of acetate contained in the hydrolysate solution will inhibit copolymer synthesis. After treating with active charcoal and ion-exchange columns to remove acetate, engineered *E. coli* BW25113 can synthesize 62.4 wt% P(5.5 mol% LA-*co*-3HB) in a medium containing the hydrolysate solution (Takisawa et al. 2017). The above conclusions all clarified the adverse effect of acetate on the copolymer production.

Although acetate inhibits copolymer synthesis, some acetate-tolerant strains are still discovered. The process of using engineered *E. coli* LS5218 along with the *pflA* gene deletion to produce the copolymers suggests that acetate played an important role in controlling LA monomer incorporation into the copolymers (Salamanca-Cardona et al. 2014a). Compared to using 20 g/L xylose alone, the overall yields of engineered *E. coli* LS5218 along with the *pflA* gene deletion increase by more than twofold with the presence of 25 mM acetate. In addition, when 25 mM acetate is used as the sole carbon source, the copolymer still can be synthesize by strain (Salamanca-Cardona et al. 2017).

High reducing power brought by anaerobic cultivation is beneficial to an increase in the flux toward LA-CoA. After a 24 h aerobic cultivation, engineered *E. coli* W3110 can synthesize 2 wt% P(47 mol% LA-*co*-3HB) when it is transferred into anaerobic conditions and is further cultured for another 24 h in a medium with 20 g/L glucose (Yamada et al. 2009). 12 wt% P(62 mol% LA-*co*-3HB) can be synthesized by combining anaerobic cultivation with engineered *E. coli* BW25113 along with the *pflA* gene deletion as well as carrying LPE of F392S mutant (Yamada et al. 2010). LA monomer fraction of the copolymers produced by engineered *E. coli* BW25113 along with the *pflA* gene deletion can be adjusted between the range from 29 to 47 mol% by the fine-regulation of the culture conditions in anaerobic cultivation with a medium containing 20 g/L glucose within a fermentation tank (Yamada et al. 2011). In addition, relatively anaerobic conditions (shaking speed = 0/60 strokes/min) can also increase LA monomer fraction in the copolymers (Goto et al. 2019b).

Fed-batch cultivation is an important technology to achieve high cell density and high volumetric productivity in a fermentation tank. By adjusting dissolved oxygen concentration (DOC) and glucose concentration (upon pH-stat feeding), LA monomer fraction can be adjusted between the range from 8.7 to 64.4 mol% by engineered *E. coli* XL1-Blue (Yang et al. 2010). Jung and Lee (2011) increase the weight percentage of P(LA-*co*-3HB) by fed-batch cultivation, but LA monomer fraction is decreased. The utilization of the pH-stat strategy or the pulsed-feeding strategy can produce aromatic PHAs to a reasonably high concentration (Yang et al. 2018). In addition, 20 g/L initial glucose concentration (0–24 h) is used for cell growth, and xylose (24–81.6 h) is used for the copolymer production, the feeding rate of the sugar solution is increased in a stepwise manner. Under this condition, engineered *E. coli* MG1655 can synthesize 44.3 wt% P(4.9 mol% LA-*co*-3HB) and the copolymer production increases significantly (Hori et al. 2019). Moreover, batch fermentation technology is used to overcome the bottleneck of the utilization of fructose, which is one of the components of rice bran (Oh et al. 2015)/sucrose (Sohn et al. 2020) hydrolysates. Compared with Pct540_*Cp*_, engineered *E. coli* XL1-Blue expressing PhaC1437_*Ps*6-19_ and Bct_*Eh*_ in batch fermentation shows higher OD_600_ and weight percentage, but LA monomer fraction is lower (David et al. 2017).

## Introduction of other monomers into lactate-based PHA

**Fig. 2:**
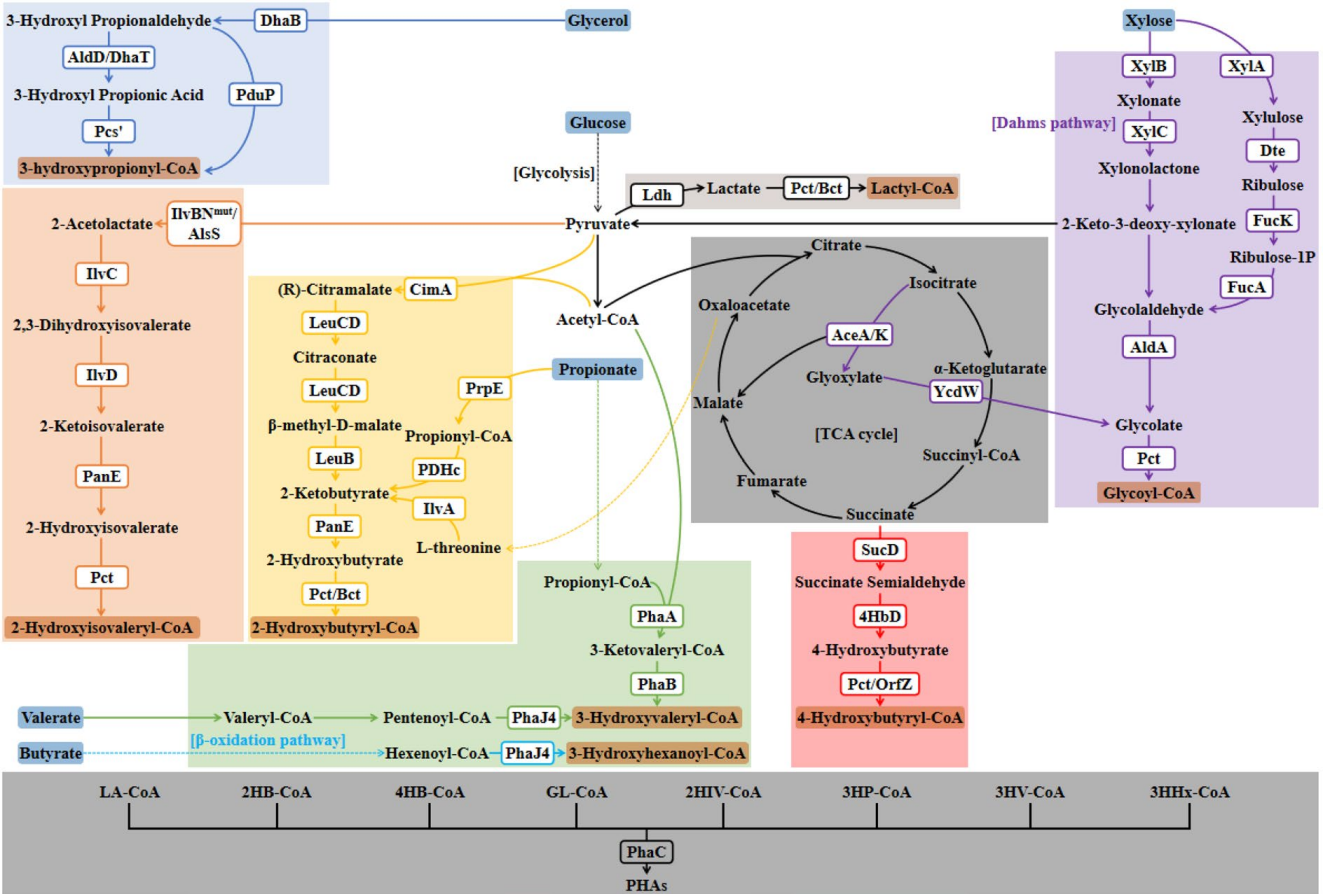
Synthetic pathway of PHA with various compositions in recombinant *E. coli*. Dotted arrows represent simplified metabolic processes. Letters in boxes indicate enzymes. *Pct* propionyl-CoA transferase, *Bct* butyryl-CoA transferase, *PhaA* β-ketothiolase, *PhaB* NADPH-dependent acetoacetyl-CoA reductase, *PhaC* PHA synthase, *Ldh* lactate dehydrogenase, *CimA* citramalate synthase, *LeuB* 3-isopropylmalate dehydrogenase, *LeuCD* isopropyl malate (IPM) isomerase, *PanE* 2HB dehydrogenase, *PrpE* propionyl-CoA synthetase, *PDHc* pyruvate dehydrogenase complex, *IlvA* l-threonine dehydratase, *SucD* succinate semialdehyde dehydrogenase, *4HbD* 4HB dehydrogenase, *OrfZ* CoA transferase, *XylB* xylose dehydrogenase, *XylC* xylonolactonase, *AceA* isocitrate lyase, *AceK* isocitrate dehydrogenase kinase/phosphatase, *YcdW* glyoxylate reductase, *XylA* xylose isomerase, *Dte* d-tagatose 3-epimerase, *FucK* ribulose kinase, *FucA* aldolase, *AldA* aldehyde dehydrogenase, *IlvBN*^*mut*^ mutant acetolactate synthase, *AlsS* acetolactate synthase, *IlvC* ketol-acid reductoisomerase, *IlvD* dihydroxyacid dehydratase, *DhaB* glycerol dehydratase, *DhaT* 1,3-propanediol dehydrogenase, *AldD* aldehyde dehydrogenase, *Pcs′* ACS domain of tri-functional propionyl-CoA synthetase, *PduP* propionaldehyde dehydrogenase, *PhaJ4* (*R*)-specific enoyl-CoA hydratase 4

Engineered *E. coli* can synthesize LA-CoA by introducing Pct, synthesize 3HB-CoA by introducing PhaAB, and synthesize the copolymers by introducing LPE. Similarly, other monomers can be introduced into the copolymers by transforming the corresponding CoA metabolic pathway into *E. coli*. Alternatively, monomers can be added to the substrate directly and then use the one-pot method to produce the copolymers (Matsumoto et al. 2013). Different monomer copolymerization strategies are summarized in Fig. 2.

### Introduction of 2HB biosynthesis pathway

The introduction of the citramalate synthase gene (*cimA3.7*) of *Methanococcus jannaschii*, the 3-isopropylmalate dehydrogenase gene (*leuB*) and the isopropyl malate (IPM) isomerase gene (*leuCD*) of *E. coli* W3110, as well as the 2HB dehydrogenase gene (*panE*) of *Lactococcus lactis* subsp. *lactis* Il1403 into *E. coli* XL1-Blue allows converting glucose into 2HB in vivo (Park et al. 2012b, 2013a; Chae et al. 2016; David et al. 2017). By introducing the propionyl-CoA synthetase gene (*prpE*) of *C. necator* and using the inherent pyruvate dehydrogenase complex (PDHc), *E. coli* XL1-Blue allows converting propionate into 2HB with the help of the *panE* gene in vivo. In addition, the deletion of the 2-methylcitrate synthase gene (*prpC*) can increase 2HB monomer fraction in the copolymers, while it will decrease the copolymer content (Park et al. 2013a). Moreover, *E. coli* can endogenously produce l-threonine, which can be transformed into 2HB with the help of the *panE* gene (Yang et al. 2016) or into 2HB-CoA with the help of the *ldhA* and *hadA* genes of *C. difficile* 630 (Mizuno et al. 2018; Sudo et al. 2020). The deletion of the l-threonine dehydratase gene (*ilvA*), will lead to the remove 2HB from the copolymers. However, since *ilvA* is the crucial gene for amino acid biosynthesis, the deletion of it in *E. coli* will result in the growth retardation and the decrease of the copolymer content. Considering the activity of l-threonine dehydratase can be inhibited allosterically by l-isoleucine, the same effect can be achieved by the strategy of adding l-isoleucine into the medium (Choi et al. 2016, 2017; Yang et al. 2016; Choi et al. 2020a). In order to increase 2HB monomer fraction, l-threonine can be added to the medium (Sudo et al. 2020). Furthermore, l-valine can also achieve the same effect, because the activity of acetohydroxy acid synthase is negatively regulated by l-valine (Yang et al. 2016; Sudo et al. 2020). In another explanation, l-valine is pointed out that allosterically activates l-threonine deaminase and catalyzes l-threonine to form 2-ketobutyrate (Sudo et al. 2020). Sudo et al. (2020) indicate that l-valine inhibits bacteria growth and leads to a decrease in the copolymer production, but this phenomenon is not discussed by Yang et al. (2016).

### Introduction of 4HB biosynthesis pathway

The introduction of the succinate semialdehyde dehydrogenase gene (*sucD*), the 4HB dehydrogenase gene (*4hbD*), and the CoA transferase gene (*orfZ*) of *Clostridium kluyveri* DSM555 into *E. coli* JM109 allows converting glucose into 4HB-CoA in vivo (Li et al. 2017). With the introduction of the *sucD* and *4hbD* genes into *E. coli* XL1-Blue and with the help of Pct540_*Cp*_, the *E. coli* mutant also allows converting glucose into 4HB-CoA in vivo (Choi et al. 2016, 2020a). In addition, the deletion of the succinate semialdehyde dehydrogenase genes (*sad* and *gabD*) can increase 4HB monomer fraction in the copolymers (Li et al. 2017). In another study, deleting the *yneI* and *gabD* genes, which coding succinate semialdehyde dehydrogenase too, can also increase 4HB monomer fraction in the copolymers (Choi et al. 2016, 2020a).

### Introduction of glycolate (GL) biosynthesis pathway

In 2011, the copolymers containing GL were synthesized in *E. coli* LS5218 for the first time with exogenous GL (Matsumoto et al. 2011). Subsequently, the methods for providing endogenous GL by modulating the metabolic pathway were established gradually. Establishing the Dahms pathway (XylBC_*ccs*_) in *E. coli* XL1-Blue by introducing of the xylose dehydrogenase gene (*xylB*) and the xylonolactonase gene (*xylC*) of *Caulobacter crescentus* allows converting xylose into GL in vivo (Choi et al. 2016, 2017, 2020a). The native glyoxylate bypass pathway of *E. coli* is amplified by the overexpression of the isocitrate lyase gene (*aceA*), the isocitrate dehydrogenase kinase/phosphatase gene (*aceK*), and the glyoxylate reductase gene (*ycdW*), which allows converting glucose into GL in vivo. The deletion of the glycolate oxidase gene (*glcD*) can increase GL monomer fraction in the copolymers (Li et al. 2016, 2017). The introduction of the d-tagatose 3-epimerase gene (*dte*) of *Pseudomonas cichorii* and the overexpression of the native genes [the ribulose kinase gene (*fucK*), the aldolase gene (*fucA*), and the aldehyde dehydrogenase gene (*aldA*)] in *E. coli* K12 also allow converting xylose into GL in vivo (Da et al. 2019).

### Introduction of other 2-hydroxyalkanoates (2HA) biosynthesis pathway

The introduction of the mutant acetolactate synthase gene (*ilvBN*^*mut*^) [or the *B. subtilis* acetolactate synthase gene (*alsS*)], the ketol-acid reductoisomerase gene (*ilvC*), and the dihydroxyacid dehydratase gene (*ilvD*) of *E. coli* W3110 into *E. coli* XL1-Blue allows converting glucose into 2-hydroxyisovalerate (2HIV) with the help of the *panE* gene in vivo. In addition, adding l-valine into the medium can also increase 2HIV monomer fraction in the copolymers (Choi et al. 2016; Yang et al. 2016). The introduction of the *ldhA* and *hadA* genes into *E. coli* DH5*α* allows converting glucose/xylose/glycerol into 2HA-CoA (2HP, 2H3MB, 2H3MV, 2H4MV, and 2H3PhP) with the supplement of different amino acids in vivo (Mizuno et al. 2018).

### Introduction of 3‐hydroxypropionate (3HP) biosynthesis pathway

The introduction of the glycerol dehydratase gene (*dhaB*) of *Klebsiella pneumoniae*, the 1,3-propanediol dehydrogenase gene (*dhaT*) and the aldehyde dehydrogenase gene (*aldD*) of *P. putida* KT2442, as well as the ACS domain of tri-functional propionyl-CoA synthetase gene (*pcs*′) of *Chloroflexus aurantiacus* into *E. coli* S17-1 allows converting glycerol into 3HP-CoA in vivo (Ren et al. 2017). The introduction of the glycerol dehydratase gene (*dhaB123*) of *K. pneumoniae* and the propionaldehyde dehydrogenase gene (*pduP*) of *Salmonella typhimurium* LT2 into *E. coli* JM109 also allows converting glycerol into 3HP-CoA in vivo (Zhao et al. 2018).

### Introduction of 3HV and medium-chain-length 3-hydroxyalkanoates (3HA) biosynthesis pathway

Using *E. coli* BW25113 along with the *pflA* gene deletion, 3HV-CoA can be supplied from propionate, which is esterified into propionyl-CoA by proposed inherent pathways (such as acetyl-CoA synthetase and propionyl-CoA synthetase) firstly. Then, Propionyl-CoA is converted into 3HV-CoA by PhaAB (Shozui et al. 2010b). The introduction of the (*R*)-specific enoyl-CoA hydratase 4 gene (*phaJ4*) of *P. aeruginosa* into *E. coli* LS5218 allows converting valerate into 3HV-CoA in vivo with the help of the β-oxidation pathway (Shozui et al. 2011). In addition, with the same strategies (using the *phaJ4* gene and the β-oxidation pathway together), *E. coli* LS5218 can polymerize 3HHx into the copolymers from butyrate (Shozui et al. 2010a) and polymerize 3HA (3HB, 3HHx, 3HO, 3HD, and 3HDD) into the copolymers from dodecanoate (Matsumoto et al. 2011). Moreover, the introduction of enzymes for the synthesis of 3HA monomers with medium-chain length into the *fadR* gene deleted *E. coli* allows converting glucose into 3HA (3HB, 3HO, 3HD, 3HDD, and 3H5DD) in vivo (Goto et al. 2019a).

## Microorganisms other than *E. coli* for P(LA-*co*-3HB) biosynthesis

Due to the relatively mature metabolic regulation mechanism and molecular tools, *E. coli* is the most widely used chassis cell in the copolymer production. While in recent years, some other non-traditional chassis cells also have been developed and applied.

As an endotoxin-free platform, a Gram-positive bacteria *Corynebacterium glutamicum* is metabolic engineered to produce the copolymers. After introducing LPE and monomer supplying enzymes, 2.4 wt% P(96.8 mol% LA-*co*-3HB) is synthesized by engineered strain in a medium with 60 g/L glucose. LA monomer fraction is further increased to 99.3 mol% after the *phaAB* gene deletion, while the copolymer content is decreased to 1.4 wt% (Song et al. 2012).

*Cupriavidus necator* is one of the most effective platforms for producing various PHAs. With the expression of LPE and monomer supplying enzymes, *C. necator* 437–540 can synthesize 33.9 wt% P(37 mol% LA-*co*-3HB) in a medium containing 20 g/L glucose. Furthermore, 2HB and 3HV can be polymerized into the copolymers by this strain when 2HB is added to the medium (Park et al. 2013b). Introducing the *sacC* and *ldhA* genes into *C. necator* 437–540 can synthesize 19.5 wt% P(21.5 mol% LA-*co*-3HB) in a medium with 20 g/L sucrose (Park et al. 2015).

Considering the higher production efficiency and the lower cost of the copolymers, *Sinorhizobium meliloti* is selected as a production platform. Pct532_*Cp*_ and PhaC1400_*Ps*6-19_ are introduced into *S. meliloti* Rm1021 and the native PHA synthase enzyme gene (*phbC*) is replaced. Under the control of the native *phbC* promoter, engineered strain can synthesize P(30 mol% LA-*co*-3HB) in a medium containing mannitol. This is the first report of the copolymer production in *Alphaproteobacteria* (Tran and Charles 2016).

## Conclusions

Due to the rising price of crude oil, the depletion of petroleum resources, and the environmental damage caused by plastic wastes, PLA and its copolymers have become the potential substitutes for the degradable synthetic plastics and the “green copolymers” made from renewable resources. Therefore, they have attracted more and more attention in the fields of industry, medicine, and research. The researches of the lactate-based copolymers, which possess broad application prospects, not only greatly improve the properties of PLA, but also greatly expand the application field of it.

It has become a trend to utilize inexpensive extraneous carbon sources (such as glucose and glycerol) and/or mixed cultures (such as agricultural wastes) in the production. Recombinant *E. coli* has been developed as a conventional platform to produce the copolymers (Choi et al. 2020b). The strategies of the copolymer production are generally to overcome the shortcomings of inducible promoters and/or introduce new key enzymes. Because of the ability of *E. coli* to use a variety of cheap and unrelated carbon sources, the large-scale bioreactors production from the culture in shake flasks should be further studied to obtain a more efficient fermentation process. In addition, the separation and the purification of the copolymers from *E. coli* is also one of the research directions.

With the further development of the potential recombinant *E. coli* strains and other related strains, it is not only helpful to expand the production types of the copolymers but also can improve the productivity and the yield of the copolymers, making copolymer recycling more convenient and economical. In order to replace the plastics derived from the petrochemical industry ideally, more researches should be conducted on reducing the production costs and improving the properties of bioplastics.

## Data Availability

Not applicable.

## Abbreviations

PLA: Polylactic acid
LA: Lactate
P(LA-*co*-3HB): Poly(lactate-*co*-3-hydroxybutyrate)
PHA: Polyhydroxyalkanoate
Ldh: Lactate dehydrogenase
Pct: Propionyl-CoA transferase
PhaA: β-Ketothiolase
PhaB: NADPH-dependent acetoacetyl-CoA reductase
PhaC: PHA synthase
3HB: 3-Hydroxybutyrate
P(3HB): Poly(3-hydroxybutyrate)
3HV: 3-Hydroxyvalerate
4HB: 4-Hydroxybutyrate
LPE: LA-CoA polymerizing enzyme
2HB: 2-Hydroxybutyrate
Bct: Butyryl-CoA transferase
FldA: Cinnamoyl-CoA:phenyllactate CoA-transferase
HadA: Isocaprenoyl-CoA:2-hydroxyisocaproate CoA-transferase
GatC: Non-ATP consuming galactitol permease
PflB: Pyruvate formate lyase
PflA: Pyruvate formate lyase activating enzyme
Mlc: Multiple regulator of glucose and xylose uptake
LDE: LA-CoA degrading enzyme
DOC: Dissolved oxygen concentration
CimA: Citramalate synthase
LeuB: 3-Isopropylmalate dehydrogenase
LeuCD: Isopropyl malate isomerase
IPM: Isopropyl malate
PanE: 2HB dehydrogenase
PrpE: Propionyl-CoA synthetase
PDHc: Pyruvate dehydrogenase complex
IlvA: l-Threonine dehydratase
SucD: Succinate semialdehyde dehydrogenase
4HbD: 4-Hydroxybutyrate dehydrogenase
OrfZ: CoA transferase
XylB: Xylose dehydrogenase
XylC: Xylonolactonase
AceA: Isocitrate lyase
AceK: Isocitrate dehydrogenase kinase/phosphatase
YcdW: Glyoxylate reductase
XylA: Xylose isomerase
Dte: d-Tagatose 3-epimerase
FucK: Ribulose kinase
FucA: Aldolase
AldA: Aldehyde dehydrogenase
IlvBN^mut^: Mutant acetolactate synthase
AlsS: Acetolactate synthase
IlvC: Ketol-acid reductoisomerase
IlvD: Dihydroxyacid dehydratase
DhaB: Glycerol dehydratase
DhaT: 1,3-Propanediol dehydrogenase
AldD: Aldehyde dehydrogenase
Pcs′: ACS domain of tri-functional propionyl-CoA synthetase
PduP: Propionaldehyde dehydrogenase
PhaJ4: (*R*)-Specific enoyl-CoA hydratase 4
GL: Glycolate
2HA: 2-Hydroxyalkanoate
2HIV: 2-Hydroxyisovalerate
2HP: 2‐Hydroxypropionate
2H3MB: 2-Hydroxy-3-methylbutyrate
2H3MV: 2-Hydroxy-3-methylvalerate
2H4MV: 2-Hydroxy-4-methylvalerate
2H3PhP: 2-Hydroxy-3-phenylpropionate
3HP: 3‐Hydroxypropionate
3HA: 3-Hydroxyalkanoate
3HHx: 3-Hydroxyhexanoate
3HO: 3-Hydroxyoctanoate
3HD: 3-Hydroxydecanoate
3HDD: 3-Hydroxydodecanoate
3H5DD: 3-Hydroxy-5-*cis*-dodecenoate
